# Primary Peritonitis Secondary to *Streptococcus pyogenes* in a Young Female Adult—A Case Report and Literature Review

**DOI:** 10.3390/idr13010005

**Published:** 2021-01-01

**Authors:** Avelyn E. Y. Aw, James W. K. Lee, Kon Voi Tay

**Affiliations:** 1 Yong Loo Lin School of Medicine, National University of Singapore, Singapore 117597, Singapore; avelyn.aw4@gmail.com; 2 Department of Surgery, National University Hospitals, Singapore 119228, Singapore; 3 Department of Surgery, Woodlands Health Campus, Singapore 768024, Singapore; kon_voi_tay@whc.sg; 4 Department of Surgery, Tan Tock Seng Hospital, Singapore 308433, Singapore

**Keywords:** primary peritonitis, spontaneous bacterial peritonitis, streptococcus pyogenes, group A streptococcus, acute abdomen, infective ascites, ascitic fluid, bacterial translocation

## Abstract

Primary spontaneous bacterial peritonitis (SBP) is a rare cause of acute abdomen in previously healthy patients, even more unusually caused by a group A Streptococcus (GAS) (also known as *Streptococcus pyogenes*) infection. We report a young, otherwise healthy female who presented with generalized abdominal pain that was initially managed conservatively as gastroenteritis, with a computed tomography (CT) scan showing a ruptured corpus luteal cyst. Upon subsequent readmission with worsened pain and symptoms, a repeat CT scan showed worsened free fluid with signs of peritonitis. A diagnostic laparoscopy confirmed primary peritonitis with an unknown infection source and causative pathology, as the appendix, ovaries and bowels were healthy-looking. Fluid cultures returned positive for GAS Pyogenes, while blood and urine cultures were negative. The discussion reviews the challenges in diagnosis and treatment of GAS primary peritonitis, highlighting the need for clinical suspicion, early diagnosis via laparoscopy or laparotomy and prompt antibiotic therapy as the current standard for treatment.

## 1. Introduction

Primary peritonitis can be defined as peritonitis in the absence of an intra-abdominal source. A usually uncommon condition, certain conditions such as immunosuppression, diabetes mellitus, HIV and chronic renal failure do confer increased predisposition [1]. Most commonly, patients present with acute onset of abdominal pain and sepsis resulting in a diagnosis of an acute abdomen and usually undergo surgical therapy. We report a case of acute abdomen with primary peritonitis secondary to group A Streptococcus (GAS) (also known as *Streptococcus pyogenes*) infection in a young female patient. In this patient demographic, there is also a need to consider toxic shock syndrome secondary to GAS infection, classically associated with tampon usage in the past [2].

## 2. Case Presentation

A 33-year-old otherwise healthy female was admitted to our hospital initially for symptoms of generalized abdominal pain for 2 days, worst in the left lower quadrant and associated with diarrhea and fever. There was tenderness on palpation associated with rebound but without guarding. Vaginal and rectal examinations were unremarkable, without any tenderness or discharge.

**Figure 1:**
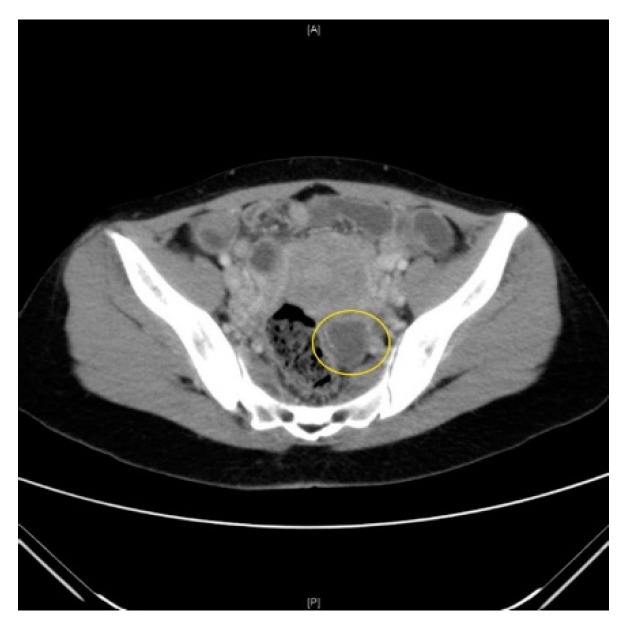
Computed tomography (CT) scan of the abdomen and pelvis with a left partially rim enhancing structure 2 × 2 cm suggestive of a ruptured corpus luteal cyst.

She was hypotensive with a blood pressure of 84/51 mmHg and tachycardic with a heart rate of 100. She was successfully resuscitated in the emergency department. Her white blood count (WBC) was raised at 15 × 10^9^ and C reactive protein (CRP) at 54 mg/L. A computed tomography (CT) scan revealed a left partially rim enhancing structure 2 × 2 cm suggestive of a ruptured corpus luteal cyst (Figure 1). There was otherwise minimal free fluid in the pelvis and no evidence of other intra-abdominal pathology. Clinically, there were no other possible sources of sepsis found to account for the presenting symptoms and signs. There were no respiratory or urinary symptoms, and the urine pregnancy test was negative.

Our patient was married with children and sexually active. Her last menstrual period was 16 days prior to this presentation, with no history of tampon or intrauterine contraceptive device (IUCD) use. She had a significant history of previous cervical dysplasia (CIN3) and had undergone treatment 5 years prior, with annual PAP smears being negative for recurrence.

She was then admitted under gynecology for further evaluation and treated with oral ciprofloxacin on account of the diarrhea with the primary diagnosis of gastroenteritis. She was discharged after 2 days with no further deterioration in her symptoms.

She was subsequently readmitted again 2 days later with recurring symptoms of similar abdominal pain that progressively worsened. She was febrile with a temperature of 38.4 °C and tachycardic with a heart rate of 133. There were no other symptoms of respiratory or urinary infection. On examination, her abdomen was guarding and exquisitely tender at all quadrants with features of generalized peritonism. No other localizing sources of infection were found on examination.

**Figure 2:**
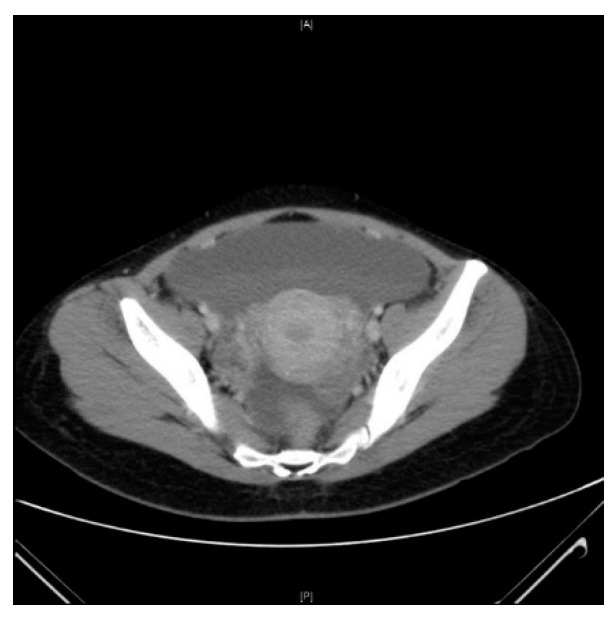
Computed tomography (CT) scan of pelvis demonstrating ascites with persistent left adnexal lesion.

Her WBC was 22 × 10^9^/L and hemoglobin (Hb) was stable at 12.8 g/dL. CRP was 108 mg/L. Liver enzymes and amylase were within normal limits. An intra-abdominal source of sepsis was suspected. A repeat CT scan was performed, which showed extensive free fluid in the abdomen suspicious for either hemoperitoneum or exudative ascites with the persistent left adnexal lesion noted from the previous study (Figure 2). No other intra-abdominal pathology was discovered on the scan with a normal appendix noted on both CT scans. In view of clinical deterioration and CT scan findings, a diagnostic laparoscopy was performed by the gynecologist on call to rule out possible ovarian cyst bleeding or ongoing intra-abdominal sepsis. Intraoperatively, we found generalized profuse seropurulent fluid. The uterus and ovaries were examined and showed no signs to suggest pelvic inflammatory disease (PID) or a bleeding cyst. On the table, a general surgery (GS) consult was made and the operation was taken over by the general surgeon. A thorough diagnostic laparoscopy was performed and the liver, spleen and rest of the alimentary tract (stomach, small and large bowels) were inspected to ensure no causative pathology. A normal appendix was identified and unlikely the cause of peritonitis, hence it was not removed. The bowels were coated with fibrin but with no sign of inflammation or perforation. In light of the unknown infection source with purulent peritonitis, abdominal drains were inserted, and the patient was continued on intravenous (IV) antibiotics in the ward.

Fluid cultures returned positive for group A Streptococcus sensitive to penicillin. Blood and urine cultures were otherwise negative. There was no history of upper respiratory tract infection (cough, sore throat and running nose) or signs of genitourinary sepsis prior to this event. A HIV screen was also done and found to be negative. A diagnosis of primary streptococcal peritonitis was made. She was treated with IV penicillin for 5 days and subsequently swapped to oral clindamycin 450 mg 3 times a day for a total duration of 2 weeks. A transthoracic echocardiogram did not show any sign of infective endocarditis. Patient showed improvement both clinically and biochemically during her stay. Drains were sequentially removed based on drain outputs with no bile or feculent discharge noted.

Patient was discharged after 10 days of treatment in the hospital and continued with oral clindamycin upon discharge as per culture sensitivities. She was subsequently reviewed by the infectious disease team in clinic in view of this atypical presentation and repeat blood tests showed the total whites and CRP had normalized. She did complain again of some lower abdominal pain which spontaneously resolved with conservative symptomatic management.

## 3. Discussion

Peritonitis can be divided into three main types: primary, secondary and tertiary, with secondary being the more common of the three. Primary peritonitis, which accounts for 1% of all peritonitis, is a very rare disease amongst individuals who are otherwise healthy and without comorbidities. It is usually associated with patients with underlying autoimmune disease such as systemic lupus erythematosus (SLE), immunosuppression, chronic liver disease, especially those with ascites, and chronic kidney disease [1]. Secondary peritonitis is frequently due to intra-abdominal lesions such as bowel perforation and ischemia. Lastly, tertiary peritonitis is characterized by persistent or recurrent infection after 48 h following successful and adequate surgical source control, usually with organisms of low intrinsic virulence, and predisposed in immunocompromised patients. It is usually associated with progressive organ dysfunction leading to high mortality [3].

The pathogenesis of spontaneous bacterial peritonitis (SBP): the translocation of gut bacteria via lymphogenous (mesenteric lymph nodes) and subsequently hematogenous spread [4].

Bacterial translocation is a known key clinical complication in patients with chronic liver disease, particularly cirrhosis. Past literature points to two main pathogenic mechanisms linking cirrhosis and SBP, namely, delayed intestinal motility and transit that is likely to lead to bacterial overgrowth; and a compromised intestinal barrier secondary to portal hypertension. Vascular stasis results in mucosal congestion and edema [5], while local hypoxia causes oxidative damage to the intestinal mucosa [6]. These events precipitate bacterial translocation into mesenteric lymph nodes.

In SLE patients, associated immune complex vasculitis and vasculopathy is known to cause ascites, then conferring a risk of spontaneous peritonitis [7]. Amongst the various proposed mechanisms of bacterial entry into the peritoneum, ascites seems to be the main commonality [6].

This phenomenon has also been observed in immunosuppressed patients, notably those on steroid therapy which has been thought to increase intestinal permeability [8], leading to translocation and hence SBP, particularly when caused by quinolone-resistant *Escherichia coli* [9]. Potential bacterial entry through skin is rare but possible, as documented in a case of a peritoneo-cutaneous fistula secondary to skin excoriation from a large, chronic incisional hernia that subsequently caused primary peritonitis [10].

However, it is unlikely that the aforementioned gut translocation mechanisms led to SBP in our patient. An ascending infection following the ruptured corpus luteal cyst possibly predisposed to the event, although other lesions such as tubo-ovarian abscesses are more commonly associated with peritonitis [11]. IUCDs and recent delivery/dilatation and curettage (D&C) procedures may precipitate an ascending infection as well.

Her initial diagnosis of gastroenteritis should not be excluded as a precipitating factor to her presentation, although gastroenteritis secondary to a GAS infection is rare. Even though a clear mechanism of infection has not been established, intestinal migration of *S. pyogenes* from a focal respiratory tract infection can result as an indirect cause of gastroenteritis [12].

One case discusses a 41 year old woman with a similar history of cervical carcinoma for which biopsies were done prior, who presented 11 years later with SBP [13]. Although in her case ascitic cultures grew *Escherichia coli* and *Enterobacter*, the actual event that precipitated her SBP was unconcluded as well.

Primary peritonitis secondary to GAS is an even more unusual occurrence, given that the organism is more commonly associated with the upper aerodigestive tract or genital urinary systems. Only a small number of cases have at present been reported in the literature, and it is interesting to note that a large majority of them were previously young healthy women with an unknown primary source as reported by Malota et al. and Litaka et al. [14,15]. The use and yield of streptococcal pharyngeal swab tests in GAS peritonitis are not reported and do not change management workflow, while the majority had positive blood cultures for *Streptococcus*. However, despite the positivity of the cultures, often patients may not present with prior symptoms of upper respiratory tract or genitourinary infections that indicate a clear source of bacteremia, which can seed into the abdominal cavity.

Various routes of inoculation have been postulated which include sources such as hematogenous, direct inoculation and translocation. Some reported skin infections from insect bites and fasciitis have been shown to cause GAS peritonitis as well in Malota et al. [14]. Rarely, cervical carcinoma can also cause peritonitis. This is speculated to occur via retrograde inoculation from the genitourinary tract [16], in which the vaginal mucosa functions as a nidus for the vascular invasion and dissemination of GAS [17]. As a complication of untreated cervical carcinoma, spontaneous pyometra rupture is reported to cause secondary peritonitis [18].

In our case, given that the source was unknown and there were no localizing symptoms for the underlying source of the bacteria, we collaborated with the infectious disease physicians for culture-directed antibiotic stewardship and further work-up to rule out distant, occult primary sources of sepsis such as infective endocarditis and septic arthritis. These also proved to be negative in our patient [19,20].

GAS infections predominantly present as respiratory, cutaneous and soft tissue infections such as pharyngitis, erysipelas, necrotizing fasciitis as well as streptococcal toxic shock syndrome (STSS). The complications of such infections vary in severity, though primary peritonitis is a rare complication [21]. Even though the patient’s blood culture returned negative, the presence of streptococcal toxin can still manifest as STSS and cause systemic upset. Patients can continue to deteriorate further without prompt diagnosis and management, eventually resulting in mortality.

**Figure 3:**
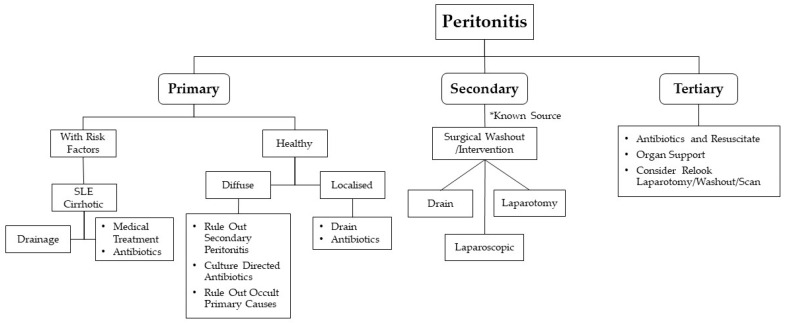
General approach to peritonitis.

Being a rare disease, the diagnosis of primary GAS peritonitis is a diagnosis of exclusion. Mainstay of management of peritonitis is as depicted in Figure 3, which includes: prompt diagnosis, early empirical antibiotic treatment and exclusion of secondary peritonitis which requires more aggressive and early surgical exploration and intervention. Active resuscitation is needed as GAS infection can present with STSS. Ultrasound and CT scan are useful diagnostic tools to look for possible underlying intra-abdominal pathology prior to surgery. A thorough physical examination needs to be carried out in patients with primary peritonitis to look for possible primary sources, e.g., throat, superficial skin infection, infective endocarditis, etc.

Diagnostic laparoscopic approach in patients with acute abdomen with an unknown primary source has gained more favor compared to conventional exploratory laparotomy. There is a need for adequate training, exposure and the promotion and support of minimally invasive approaches initiated from advanced to junior surgical trainees for application in acute abdomen cases that involve investigating for septic foci [22].

It is prudent to ensure adequate resuscitation prior to laparoscopic approach, as the pneumoperitoneum might worsen the patient’s hemodynamic stability. In the case of a negative diagnostic laparoscopy, where a primary source remains unidentified, Cortese et al. corroborates on the role of sending intraperitoneal fluid found intra-abdominally for culture [23]. In the reported case, the intra-abdominal ascitic fluid returned positive for *Streptococcus pneumoniae*. The case report and series of literature reviews of primary peritonitis from *Streptococcus pneumoniae* demonstrated similar propensity in the presentation and demographic distribution as in GAS peritonitis.

It is pivotal for the surgeon to ensure thorough examination of all organs. At the end of surgery, drains are commonly placed to drain out the washout fluid and to look for delayed perforation of the bowels in sealed perforation cases. If the patient remained unstable, conventional midline laparotomy is the gold standard of care.

There are also studies that expound on mutations in the csrS, csrR and rgg genes–negative regulator genes of GAS virulence as crucial factors in the pathogenesis of STSS [24,25], and may perhaps harbor potential causes of GAS peritonitis. We consider Kaneko’s et al. reported case, in which a 28 year-old healthy female patient developed progressive abdominal symptoms with proven GAS bacteremia, on a background of a csrS gene mutation [26]. This raises the possible need for further studies with mutation mapping of these particular virulent strains, along with new testing kits in order to tackle this group of patients [27].

## 4. Conclusions

Primary GAS peritonitis is still considered a very rare condition. Clinical suspicion and early diagnosis via laparoscopy or laparotomy are important for patients who present with symptoms and signs of an acute abdomen, as well as those who were initially treated conservatively but failed to show improvement. Mainstay of treatment is still antibiotic therapy for the underlying infection. It is very important to examine the patient carefully for potential occult primary sources of the infection to ensure the patient is treated appropriately with adequate source control.

## Data Availability

No new data were created or analyzed in this study. Data sharing is not applicable to this article.
